# Epigenetic and genetic dissections of UV-induced global gene dysregulation in skin cells through multi-omics analyses

**DOI:** 10.1038/srep42646

**Published:** 2017-02-17

**Authors:** Yao Shen, Milda Stanislauskas, Gen Li, Deyou Zheng, Liang Liu

**Affiliations:** 1 Department of Systems Biology, Columbia University, New York, NY, USA; 2 Department of Dermatology, Columbia University, New York, NY, USA; 3 Department of Biostatistics, Columbia University, New York, NY, USA; 4 Department of Genetics, Albert Einstein College of Medicine, New York, NY, USA; 5 Department of Neurology, Albert Einstein College of Medicine, New York, NY, USA

## Abstract

To elucidate the complex molecular mechanisms underlying the adverse effects UV radiation (UVR) on skin homeostasis, we performed multi-omics studies to characterize UV-induced genetic and epigenetic changes. Human keratinocytes from a single donor treated with or without UVR were analyzed by RNA-seq, exome-seq, and H3K27ac ChIP-seq at 4 h and 72 h following UVR. Compared to the relatively moderate mutagenic effects of UVR, acute UV exposure induced substantial epigenomic and transcriptomic alterations, illuminating a previously underappreciated role of epigenomic and transcriptomic instability in skin pathogenesis. Integration of the multi-omics data revealed that UVR-induced transcriptional dysregulation of a subset of genes was attributable to either genetic mutations or global redistribution of H3K27ac. H3K27ac redistribution further led to the formation of distinctive super enhancers in UV-irradiated cells. Our analysis also identified several new UV target genes, including *CYP24A1, GJA5, SLAMF7* and *ETV1*, which were frequently dysregulated in human squamous cell carcinomas, highlighting their potential as new molecular targets for prevention or treatment of UVR-induced skin cancers. Taken together, our concurrent multi-omics analyses provide new mechanistic insights into the complex molecular networks underlying UV photobiological effects, which have important implications in understanding its impact on skin homeostasis and pathogenesis.

Gene and environment interactions play pivotal roles in human disease pathogenesis and etiology. Skin serves as the major barrier structure between the body and the environment to protect the body from environmental stressors. Skin has also been shown to function as a peripheral neuroendocrine organ that regulates both local and global homeostasis through its melatoninergic system^1^,^2^, steroidogenic system^3^, and a peripheral equivalent of the hypothalamus–pituitary–adrenal (HPA) axis^4^. The epidermis of the skin interfaces directly with the outside environment. This strategic location makes the epidermis an ideal *in vivo* model organ for studying the mechanisms underlying gene and environment interactions in development and human diseases. Frequent exposure of the epidermis to environmental carcinogens greatly increases the risk and incidence of skin cancers, including both melanoma and non-melanoma skin cancers. In fact, skin cancers are the most common cancer in the United States, affecting more people than all other cancers combined^5^,^6^, which underscores the adverse effects of direct exposure to environmental carcinogens in human health and cancer susceptibility.

Solar UV radiation (UVR) is an established environmental carcinogen in skin tumorigenesis. Excessive exposure to solar UVR, particularly its UVB component, can cause a variety of harmful effects on human skin including sunburn, photoaging, immune suppression, and increased susceptibility to cancers^7^,^8^. The skin pigmentary system serves as the primary defense against the harmful effects of UVR^9^. The secosteroids produced by epidermal keratinocytes can also protect against the DNA damaging effects of UVB radiation^10^. Furthermore, UVR may alter whole-body homeostasis via activation of the skin HPA axis to increase serum levels of corticosterone^11^. At the molecular level, UV can exert its harmful effects via DNA damage, epigenetic lesions, and dysregulated gene expression. While each of these events may arise independently, they may also impinge on each other in response to UVR. The mutagenic effects of UV have been studied extensively and the mechanisms are relatively well characterized^12^,^13^,^14^. In contrast, the impact of UV on the epigenome and its contribution to transcriptome regulation remain poorly understood. Recent DNA methylomics studies have provided some preliminary but interesting insights into how chronic solar UVR may contribute to skin photoaging via aberrant DNA methylation^15^,^16^. However, repeated exposures of normal human skin cells to low doses of UVR have no recognizable effects on global DNA methylation^17^. Additional studies are needed to further elucidate the role of epigenetic mechanisms underlying the pathophysiological impact of UVR in the skin.

We and others have reported previously that acute UV exposures can cause substantial transcriptomic instability affecting thousands of genes^18^,^19^,^20^. Our recent RNA-seq studies have generated a large cohort of UV-responsive transcriptomic data using keratinocytes from different genetic background^21^. Furthermore, meta-analysis of the transcriptomic cohorts reveals that UV-induced changes in the transcription of a subset of genes are highly conserved and persistent over time^21^. These findings prompt us to test whether UV may induce genetic and/or epigenetic changes to cause persistent target gene dysregulation.

In this study, we performed concurrent RNA-seq, exome-seq, and H3K27ac (histone 3 lysine 27 acetylation) ChIP-seq studies to simultaneously characterize UV-induced genetic, epigenetic, and transcriptional changes in isogenic human keratinocytes under identical UVR experimental settings. We then performed bioinformatics and statistical analyses on the resulting omics data to decipher the interactions among the genome, epigenome and transcriptome following UVR. These analyses provide new molecular insights into the complex interactions between UV and skin cells. Furthermore, comparison of the UV gene expression signature with a human squamous cell carcinoma (SCC) signature identifies several novel UV target genes for developing targeted prevention and therapy of UV-induced skin cancers.

## Results

### Multi-omics analysis of UV-induced molecular abnormalities

**Figure 1:**
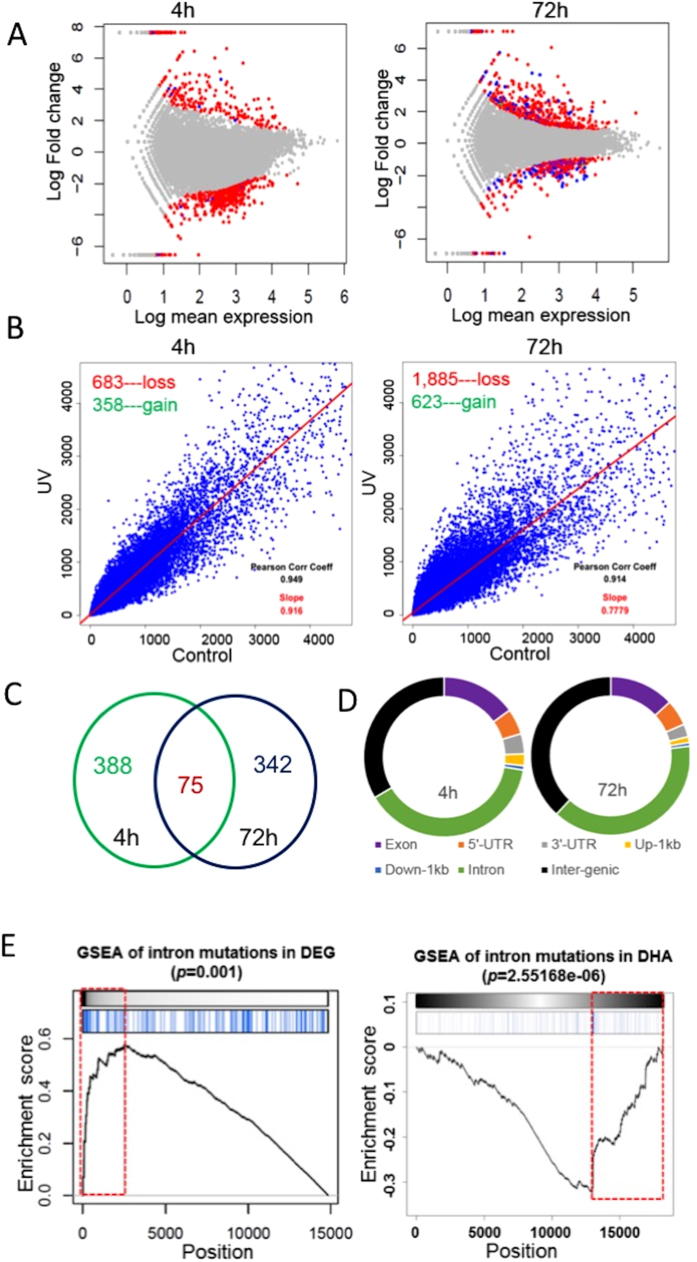
(**A**) Differential gene expression plots demonstrating transcriptomic changes in human keratinocytes following UVR. Each red dot represents a DGE 4 h or 72 h following UVR. Each blue dot represents a DGE that also displays differential H3K27 acetylation following UVR; (**B**) UV induced progressive losses of H3K27ac in human keratinocytes at 4 h and 72 h after UVR. x/y-values are tag numbers in merged peak regions. Slope value <1 indicates a net loss of H3K27ac; (**C**) Venn diagram showing that 75 SNVs are common between the 4 h and 72 h SNV sets; (**D**) A schematic illustration of genomic distributions of UV-induced SNVs at 4 h and 72 h after UVR; (**E**) GSEA analysis showing that genes containing intron mutations are significantly enriched in the DGE gene set (left panel) or DHA gene set (right panel) as highlighted by the red dotted rectangles. GSEA was based on the Kolmogorov-Smirnov test. The p-values were estimated from permutation tests by randomly shuffling genes.

The mutagenic and transcriptional effects of UV have been studied extensively in the past, but relatively few studies have investigated the impact of UV on the epigenome. H3K27ac is an epigenetic mark that is frequently present at promoters or enhancers, which also separates active enhancers from poised enhancers^22^,^23^,^24^. To test whether UV-induced differential gene expression (DGE) may be functionally linked with differential H3K27 acetylation (DHA), we performed parallel RNA-seq and ChIP-seq studies to profile global DGE and DHA in UV-irradiated human keratinocytes. As shown in Fig. 1A, UV induced substantial transcriptomic changes as highlighted in the DGE plots by red or blue dots (representing significant DGEs, p < 0.05). Similarly, ChIP-seq analysis revealed that UV caused a genome-wide loss of H3K27ac with regional gains in H3K27ac levels (Fig. 1B, slope value <1). To isolate genes associated with DHA, we calculated the FC between the average peak value of H3K27ac peaks assigned to a specific gene (within 10 kb of the start or end of a nearby gene) in the UV-irradiated sample and that in the control sample. DHA was defined using a FC cutoff at 2. Altogether, we obtained 1,041 DHA genes at 4 h and 2,508 DHA genes at 72 h following UVR, suggesting a progressive genome-wide redistribution of H3K27ac marks. Genes with significant changes in both mRNA expression (DGE) and H3K27ac (DHA) are highlighted in blue in the DGE plots in Fig. 1A.

In addition to DGE and DHA analyses, we performed concurrent WES studies using cells from the same experiment. Mutation calling using the Samtools program identified 463 and 417 single nucleotide variations (SNVs) at 4 h and 72 h (Fig. 1C, and Supplementary Tables 1 and 2), respectively, revealing a relatively moderate mutagenic effect compared to the substantial changes in global gene expression and H3K27ac in response to UVR. There were 75 common SNVs between the 4 h and 72 h mutation profiles, with 54 of them mapped within or near genes (26 in introns, 15 in exons, 2 in the 3’-UTR, 9 in the 5’-UTR, 2 in 1 kb upstream, Supplementary Table 3), and 21 in intergenic regions. Genomic distribution of UV-induced SNVs is schematically illustrated in Fig. 1D. Overall, SNVs mostly occurred in introns and intergenic regions, followed by exons, 5’-UTR, 3’-UTR, and 1 kb upstream or downstream of the genes.

Accumulating evidence supports the role of introns in regulating gene expression through cis-acting elements^25^,^26^,^27^. The predominant distribution of SNVs in introns and intergenic regions indicated that UV-induced mutations might alter gene activities transcriptionally. Indeed, GSEA analysis revealed that genes with intronic mutations were significantly enriched in the DGE list at 72 h after exposure (p = 0.001, Fig. 1E, left panel). Among them, CYP24A1 was dramatically upregulated by UVR (Log_2_FC = 7). CYP24A1 is an enzyme that can metabolize vitamin D_3_ to generate biologically active hydroxyderivatives with efficient anti-tumorigenic activities on melanoma cells. Elevated levels of CYP24A1 are associated with increased aggressiveness and proliferative potential of colorectal and prostate tumors^28^,^29^. Besides the effect of intronic mutation on gene expression, GSEA also revealed a significant overlap between genes with intronic mutations and genes showing reduced H3K27ac marks (p = 2.6e-06, Fig. 1E, right panel), consistent with the accumulating evidence supporting the role of chromatin conformation in modulating DNA repair activity during UV-induced mutagenesis^14^,^30^.

### UV induced dynamic reorganization of super enhancers (SEs)

**Figure 2:**
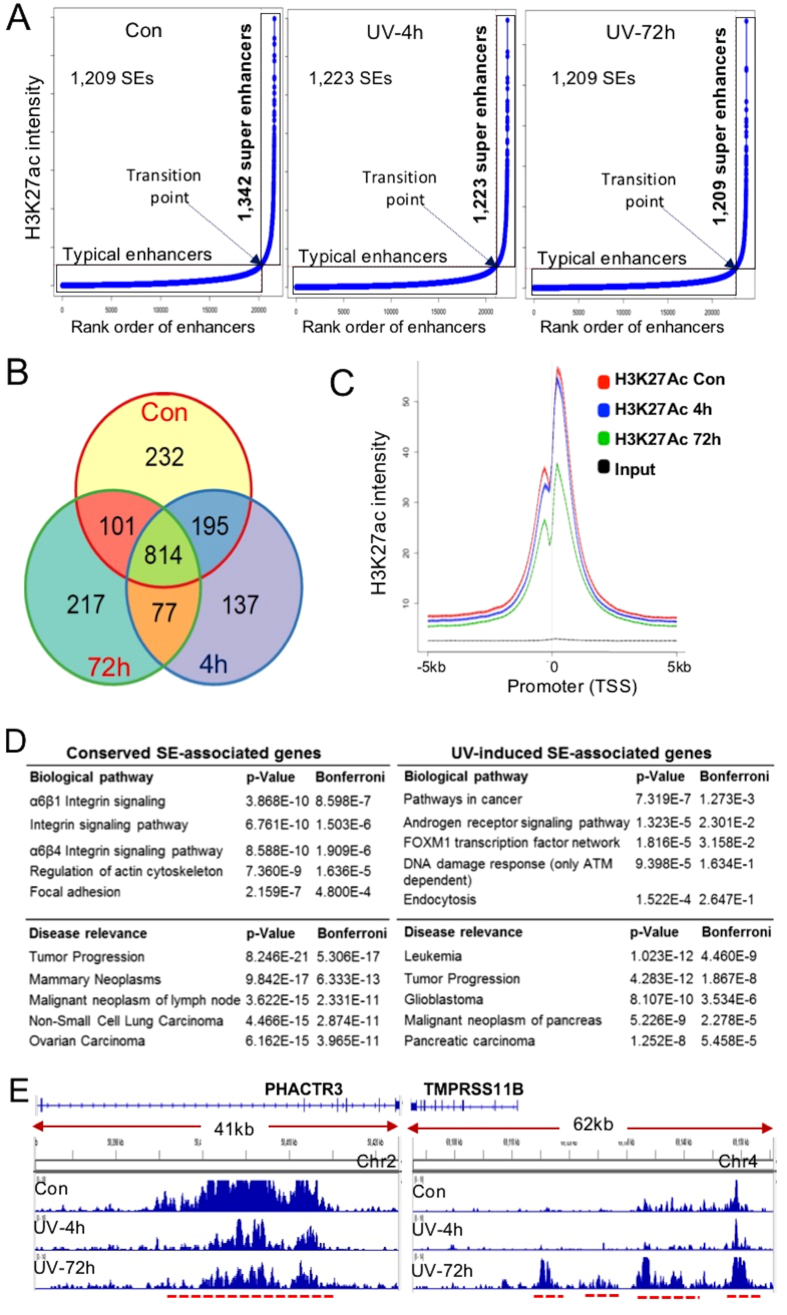
(**A**) SE profiles in control and UV-irradiated keratinocytes showing that UV decreased the total number of SEs marked by H3K27ac; (**B**) Venn diagram showing the number of common and distinctive SEs among control, UV-4h, and UV-72h; (**C**) Genome-wide H3K27ac signals in promoter regions showing a pronounced loss of 72 h following UVR; (**D**) Top biological pathways and relevant disease pathways in which the conserved SE-associated genes or UV-induced SE-associated genes are enriched. P-values were obtained using the hypergeometric distribution test to examine the overlap between the identified gene sets and the known pathways. Bonferroni correction was used to have adjusted p-values; E: Gene tracks of H3K27ac ChIP-seq exemplifying that UVR increased H3K27ac at the PHACTR3 gene locus but reduced H3K27ac at the TMPRSS11B gene locus. PHACTR3: phosphatase and actin regulator 3; TMPRSS11B: transmembrane protease, serine 11B (HATL5).

SEs are large clusters of enhancers that regulate the activity of key genes during development and disease pathogenesis^31^,^32^. H3K27ac is one of the best characterized epigenetic marks for mapping genome-wide SE structures^33^,^34^. To test whether UVR may alter SEs to modulate its target gene activities, we used the ROSE algorithm to map SEs in both control and UV-irradiated keratinocytes. We sorted the enhancer regions based on their H3K27ac signals from the lowest to the highest. Enhancers whose signals were higher than the transition point of the curve (Fig. 2A) were designated as SEs. A total of 1,342 SEs were identified in control keratinocytes. Following UV irradiation, the total number of SEs decreased to 1,223, and 1,209 SEs at 4 h and 72 h after exposure, respectively (Fig. 2A), revealing a net loss of SEs following UVR. Venn diagram in Fig. 2B illustrates that UV induced 214 unique SEs at 4 h, and 294 unique SEs at 72 h after UV exposure, with 77 UV-specific SEs conserved between the 4 h and 72 h SE sets. The majority of the SEs in non-irradiated cells (814 out of 1,342), however, remained intact after UVR. Separate analyses further revealed that UVR also decreased global H3K27ac signals at promoter regions (Fig. 2C).

Next, we isolated genes associated with either the common SEs or UV-induced SEs as indicated in Fig. 2B. We used the ToppGene Suite program to identify top biological pathways in which each group of SE-associated genes were enriched. As summarized in Fig. 2D, many of the SE-associated genes play important roles in tumorigenesis. The common SE-associated genes were enriched in integrin-dependent signaling pathways, which are essential in epidermal development and homeostasis^35^,^36^. In contrast, genes associated with UV-induced SEs were enriched in cancer-, DNA damage-, and endocytosis-related pathways (Fig. 2D). Examples of UV-induced changes in SEs are shown in Fig. 2E, where UV reduced H3K27ac signal of the SE associated with *PHACTR3* but increased H3K27ac signal of the SE associated with *TMPRSS11B*. DNA hypermethylation of *PHACTR3* is frequently observed in HPV-induced immortalization of keratinocytes and in human cancers^37^,^38^, highlighting the importance of epigenetic regulation of its activity in human diseases.

### Functional associations between global H3K27ac and gene expression regulation

**Figure 3:**
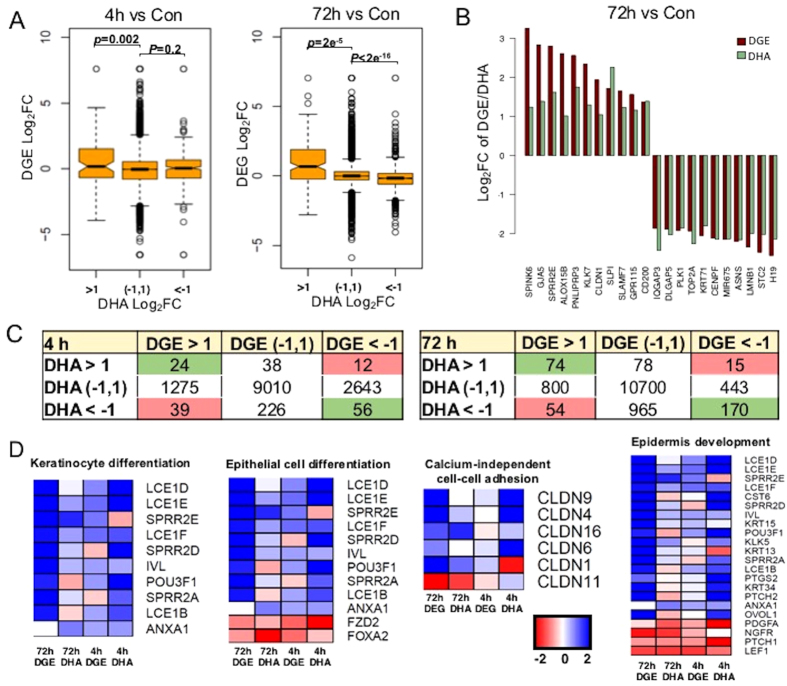
(**A**) Integrative analyses of the DGE and H3K27ac DHA gene sets at 4 h or 72 h after UVR. Correlations between gene expression and H3K27ac are considered significant if p < 0.05. P-values were obtained using Student’s t-test by comparing the log2FC of the expression values of the genes from the three DHA groups; (**B**) Representative genes showing concordant changes in gene expression and H3K27ac following UVR. Cutoff is set at Log_2_FC > 1 or <−1 for both DGE and DHA; (**C**) A summary of the overall correlations between DGE and DHA changes among UV-responsive genes at 4 h or 72 h after UVR. Pink highlights positive correlations; green highlights inverse correlations between DGE and DHA; (D) Parallel analysis of H3K27 DHA status of the DGEs that are enriched in top UV-responsive biological pathways.

To test the impact of H3K27ac redistribution on transcriptome dysregulation following UVR, we divided DHA gene set and DGE gene set into three groups based on their respective Log_2_FC values, including Log_2_FC > 1, Log_2_FC < −1, or −1 < Log_2_FC < 1 (which was considered less or non-responsive to UVR). We plotted UV-induced DGE set against DHA set at 4 h or 72 h using the R software package. As shown in Fig. 3A, we found significant correlations between genes showing increased H3K27ac (Log_2_FC > 1) and upregulated expression at both 4 h and 72 h after UVR. In contrast, significant correlations existed between decreased H3K27ac (Log_2_FC < −1) and reduced gene expression only at 72 h but not 4 h after UVR, suggesting a time-dependent effect on H3K27ac change on gene expression regulation.

Representative genes with concordant changes in gene expression and H3K27ac are shown in Fig. 3B. Genome-wide associations between H3K27ac and gene expression of UV target genes are summarized in Fig. 3C, where positive correlations are highlighted in pink and inverse correlations are highlighted in green. The majority of the UV-responsive genes displayed discordant changes in H3K27ac and expression regulation. DAVID Pathway analysis of the UV target genes using the DAVID program identified top-ranked UV-responsive pathways including keratinocyte differentiation, epithelial cell differentiation, calcium-independent cell-cell adhesion, and epidermal development (Fig. 3D). A parallel H3K27ac analysis of the genes involved in these pathways demonstrated, however, the regulation of their gene expression was largely independent of H3K27ac changes, suggesting that other transcription regulatory mechanisms were involved to alter UV target gene expression.

### UV-responsive TF motifs and target genes in skin cancer cell growth and survival

**Figure 4:**
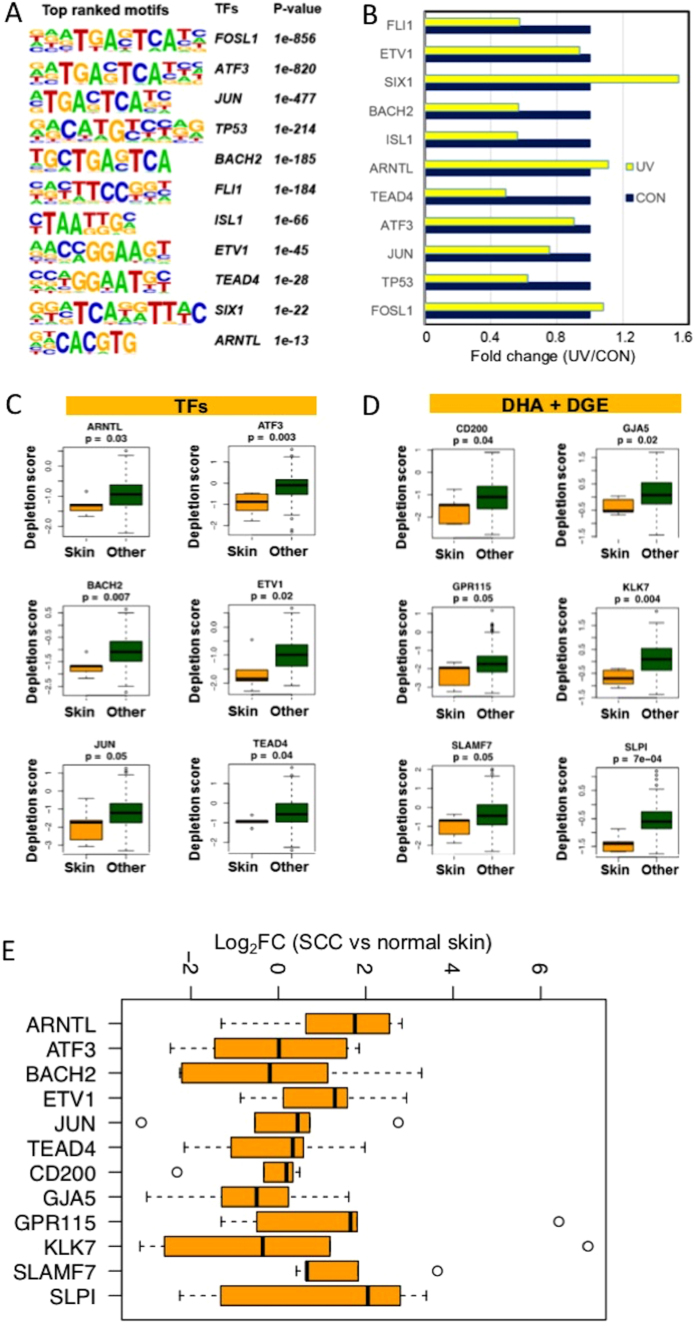
(**A**) Motif analysis showing a significant enrichment of multiple TF motifs in UV-induced DHA regions in keratinocytes following UVR; (**B**) RNA-seq results showing mRNA expression changes of the TFs identified in Fig. 4A between UV-irradiated and control keratinocytes; (**C**) Loss of function of selected UV-responsive TFs is significantly more detrimental to skin cancer cells than non-skin cancer cells; (**D**) Loss of function of selected UV target genes in Fig. 3B (more than 2-fold increases in both DGE and DHA) is significantly more detrimental to skin cancer cells than non-skin cancer cells. P-values were obtained using the Wilcoxon test by comparing the gene depletion scores between the skin cancer cells versus the non-skin cancer cells; (**E**) Box plot illustrating the Log_2_FC in the expression of the genes shown in Fig. 4C and D among 5 pairs of SCC and normal skin tissues. SLAMF7, ARNTL, ETV1, and GPR115 show more consistent upregulation in SCCs.

Previous chromatin accessibility analysis shows that UV can induce genome-wide chromatin compaction^39^, which coincides with the global loss of H3K27ac after UVR. To test whether UVR-induced changes in chromatin accessibility may occur at TF binding sites, we performed TF motif analysis focusing on H3K27 DHA regions using the HOMER algorithm. We found a significant enrichment of multiple TF motifs occurred at UV-induced DHA regions (Fig. 4A), suggesting that binding of these TFs was modulated by UVR. The majority of the identified UV-responsive TFs, such as JUN, TP53 and FOSIL1, showed moderate changes in their mRNA levels (Fig. 4B). They may contribute to the differential expression of UV target genes through chromatin accessibility changes after UVR.

Project Achilles focuses on identifying genetic vulnerabilities and generating high quality gene essentiality datasets and rigorous analytical tools. The Achilles database consists of experimental data on the function of selected genes in cancer cell growth and/or survival based on genome-wide shRNA screenings studies^40^. To test the role of UV-responsive TFs in skin carcinogenesis, we queried the Achilles database for experimental evidence on which TFs are critical to skin cancer cell growth and survival. As shown in Fig. 4C, shRNA-mediated knockdown of 6 UV-responsive TFs were significantly more toxic for cutaneous melanoma cells (A2058, C32, HS944T, SKMEL5) than other types of cancer cells (p < 0.05). Similarly, we queried the Achilles database to test the role of UV target genes in skin cancer growth and survival. We found multiple UV target genes to be critical to the survival of skin cancer cells, including CD200, GJA5, GPR115, KLK7, SLAMF7 and SLP1 (Fig. 4D).

Given the lack of RNA-seq data on cutaneous SCCs in The Cancer Genome Atlas (TCGA), we performed RNA-seq studies on 5 pairs of cutaneous SCC tumors and matched adjacent normal skins to generate a SCC-specific DGE cohort containing genes that were dysregulated in SCCs. We then queried this SCC DGE cohort to determine the expression of the UV-responsive TF genes and UV target genes shown in Fig. 4C and D. As illustrated in Fig. 4E, many of these TF genes and UV target genes displayed individual variations in their DGE status among the SCC patients. SLAMF7, ARNTL, ETV1, and GPR115 were consistently upregulated in SCCs and in response to UVR, whereas GJA5 was frequently down-regulated in SCCs but upregulated by UVR in keratinocytes.

### Validation of selected UV target genes in human SCCs

**Figure 5:**
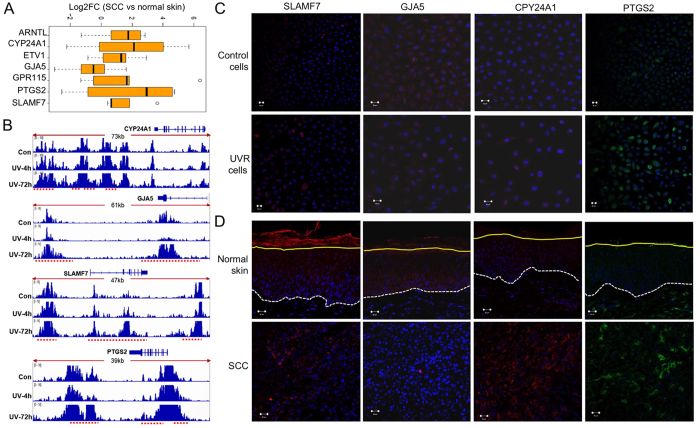
(**A**) Box plot illustrating the Log_2_FC in the expression of selected UV target genes between the 5 matched pairs of SCC and normal skin tissues; (**B**) Gene tracks of H3K27ac profiles showing that UVR increased H3K27ac levels at CPY24A1, PTGS2, GJA5, and SLAMF7 chromatin regions 72 h after UVR, which are highlighted by red dotted lines under each gene track; (**C**) Immunofluorescence staining showing protein expression of selected UV target genes in UV-irradiated keratinocytes; (**D**) Immunofluorescence staining showing protein expression of selected UV target genes in matched human SCC tumors and adjacent normal skin tissues. Blue: DAPI staining. Basement membrane in the normal skin is highlighted by the white dotted line. The stratum corneum is separated by the yellow line. Scale bar = 20 μm.

Comparison of the UV gene expression signature derived in keratinocytes with the SCC signature revealed numerous UV target genes to be consistently dysregulated in human SCCs. mRNA expression changes of selected UV target genes in SCCs relative to matched normal tissues are shown in Fig. 5A. ChIP-seq profiles at these selected gene loci demonstrated that UV induced pronounced increases in H3K27ac 72 h after UVR (Fig. 5B), consistent with the upregulation of their mRNA expression by UVR. By immunofluorescence staining, we confirmed that protein expression of SLAMF7, GJA5, CYP24A1 and PTGS2 were all elevated in UV-irradiated keratinocytes (Fig. 5C). PTGS2 is a well-characterized UV target gene that is frequently upregulated in skin carcinogenesis^41^,^42^. Next, we performed immunofluorescence staining to compare the protein expression of the UV target genes between SCC tumors and normal skins. We found that PTGS2, SLAMF7, and CYP24A1 protein levels were elevated in human SCC tissues, but GJA5 was decreased in SCCs (Fig. 5D). SLAMF7 is an established therapeutic target for multiple myeloma, and a monoclonal antibody (elotuzumab) targeting SLAMF7 can activate natural killer cells to selectively kill myeloma cells^43^. The GJA5 protein is a component of gap junctions. The biological significance of its inverse regulation by UVR and in SCCs awaits further investigations. CYP24A1 mRNA expression is elevated in multiple malignancies. In addition to the UV-induced mutation in CYP24A1 intron, increased H3K27ac may also contribute to its aberrant upregulation in skin SCC.

## Discussion

Elucidating the complex molecular mechanisms underlying UV-gene interaction will offer new insights into how UVR modulates skin homeostasis and disease pathogenesis to help improve the prevention of UV-induced skin diseases. Our study represents the first concurrent multi-omics analysis of UV interactions with the genome, epigenome and transcriptome using isogenic cells from the same UV experiment, which minimizes genetic and experimental variations. While our analysis reveals a positive functional correlation between DHA and DGE among a subset of UV target genes, the majority of the UV target genes display discordant changes or, in some cases, inverse correlations between DHA and DGE after UVR (Fig. 3C), suggesting that H3K27ac alone is insufficient to predict gene expression. UV may cause other epigenetic changes such as DNA methylation and differential histone modifications to dynamically modulate its target gene activity. In this study, we focused on H3K27ac mainly because it is one of the best-characterized epigenetic marks associated with active enhancer and promoter regions^22^,^44^. The open chromatin regions marked by H3K27ac may be indicative of frequent binding of transcription factors. The ultimate outcome of gene expression regulation may be co-determined by a combination of other histone modifications including acetylation of H3K9 and H3K18 45,46, or methylation of H3K4me1/3, H3K9me3, H3K27me3 that are linked with either active or poised enhancers and promoters^23^,^47^,^48^. The diverse repertoire of histone modifications together with their interacting regulatory proteins underscore the importance and need of systematic omics-based studies to better understand the mechanisms underpinning UV-gene interactions in skin disease pathogenesis.

UV irradiation is a primary risk factor for both melanoma and non-melanoma skin cancers^49^,^50^. Excessive exposure to solar UVR can cause cumulative genetic and epigenetic damages that disrupt gene expression preceding malignant transformation in sun-exposed skin areas. We have validated that some of the novel UV target genes discovered by our RNA-seq studies are dysregulated in human SCCs, which may also have important implications for melanomagenesis. CYP24A1, for example, is an enzyme that can metabolize vitamin D3 to generate biologically active hydroxyderivatives of 20(OH)D3, which possesses efficient anti-tumorigenic activities on melanoma cells^51^. Paradoxically, elevated levels of CYP24A1 have been reported in melanocytic nevi and early stage melanomas, highlighting the complex role of CYP24A1 in skin tumorigenesis^52^. SLAMF7 is a receptor present on immune cells including natural killer (NK) cells that mediates inhibition of NK cells in the absence of EAT-2. Elotuzumab, a monoclonal antibody targeting SLAMF7, has been approved recently as an immunotherapy agent for treating multiple myeloma^43^. SLAMF7 expression is undetectable in normal skin. SLAMF7 mRNA and protein levels are elevated in a subset of human melanoma tissues (data from The Cancer Genome Atlas and The Human Protein Atlas), making SLAMF7 an attractive immunotherapeutic target in for treating SLAMF7-positive melanoma patients.

UV-induced epigenetic effects via H3K27ac may persist in UV-irradiated cells and contribute to the malignant transformation of UV-damaged cells over time. While regional gains of H3K27ac occur following UVR, UV induces progressive global losses of H3K27ac that are especially pronounced at 72 h after exposure (Figs 1B and 2C). The genomewide loss of H3K27ac may be due to suppressed histone acetyltransferases (HATs) activities^53^, while the regional gain in H3K27ac may occur due to the binding of UV-responsive TFs such as JUN/FOS or TP53 that in turn recruits HATs to their target regions. A survey of mRNA expression of 17 HATs and 18 histone deacetylases (HDACs) based on the RNA-seq results reveals an initial downregulation of HAT members (CLOCK, KAT6, KAT7 and NCOAs) and HDAC members (HDAC4, HDAC7, HDAC9, SIRT1) at 4 h after UVR (Supplemental Table 2). By 72 h, however, there are no pronounced changes in mRNA levels of either HATs or HDACs except a 2.9-fold increase in SIRT4 (Supplemental Table 4). SEs are crucial regions of the genome consisting of clusters of enhancer elements that are enriched in H3K27ac and TFs. Despite the dynamic H3K27ac redistribution, the amount of SEs defined by H3K27ac signal peaks following UVR remains relatively stable. Pathway analyses of genes associated with common SEs in control and UV-irradiated keratinocytes reveal a significant enrichment of genes in epidermal development and function. In contrast, genes associated with UV-induced SEs are enriched in pathways of DNA damage response (CDKN1B, TP73, CDC42), consistent with the proposed function of SEs in the regulation of cell identity and state^54^.

Our concurrent omics analyses also show that the mutagenic effect of UV is relatively moderate compared to the extensive epigenomic and transcriptomic changes affecting thousands of genes. While WES is primarily used to identify mutations in coding regions, WES also generates high-quality sequence reads from noncoding regions including introns, UTRs, and intergenic regions^55^,^56^. Our study reveals that approximately 13% of UV-induced SNVs are located in exons, whereas the rest are found in introns or intergenic regions (Fig. 1C). While mutations in protein-coding regions have been the primary focus in disease research, there are growing interests in understanding the role of non-coding mutations after multiple studies demonstrating that the overwhelming majority of mutations, both somatic and germline, occur in non-coding portions of the genome. Our GSEA analysis identifies a significant correlation between UV-induced intron mutations with both DGE and H3K27ac DHA (Fig. 1E), indicating that intron mutations may interact with the epigenetic machinery in gene regulation. The C to G mutation at the Chr20:52789743 site in the CYP24A1 intron is within a region containing the binding sites of multiple chromatin modifiers such as EZH2, RBBP5, and USF1, highlighting the potential role of this CYP24A1 mutation in its expression regulation.

Our WES analysis demonstrates that C > T/G > A are the most common UV-induced SNVs (Supplemental Table 5), consistent with the UV signature mutation as seen in skin cancers^57^,^58^,^59^. The percentage of C > T mutations identified in our WES analysis, however, is lower than the percentage observed in skin cancers. The discrepancy may be due to that the mutation profile discovered in our study represents the effect of one single UV exposure event, whereas the mutation profiles in skin tumors reflect long-term cumulative effects of UV exposures. In support of this possibility, the UV-induced mutation profile in our study is highly similar to the one observed in mouse melanomas that are induced by one single neonatal UV exposure^57^.

In summary, our concurrent multi-omics studies provide new insights into the complex molecular mechanisms underlying UV photobiological effects, which have important implications in understanding its impact on skin homeostasis and disease pathogenesis. Our analysis also identified several new UV target genes, including *CYP24A1* and *SLAMF7*, which are aberrantly expressed in human SCCs. The new UV target genes and UV-responsive TFs that we have identified have important clinical implications in skin carcinogenesis, making them attractive targets for developing novel approaches for skin cancer prevention and treatment.

## Material and Methods

### Human keratinocytes, SCC tissues and adjacent normal skin tissues

Primary human keratinocytes from a neonatal foreskin (Caucasian donor) were obtained through the Columbia University Skin Disease Research Center (SDRC) Tissue Culture Core facility as described previously^20^. The SDRC routinely collects neonatal foreskins from healthy newborns through the Children’s Hospital at Columbia University Medical Center (CUMC) under an IRB protocol (# AAAD6866) that was approved by the CUMC Institutional Review Board. All foreskin samples were de-identified prior to being received by researchers and designated as non-human subject research under 45 CFR Part 46. UV radiation was supplied by 4 FS20T12/UVB tubes (National Biological Corp., Beachwood, OH), which emit UV rays between 290 and 340 nm with 75% emission in the UVB, and 25% emission in the UVA spectra, with an emission peak at 313 nm wavelength^20^,^60^. The UVR dose was measured using an IL1700 radiometer and a SED240 UVB detector (International Light, Newburyport, MA) at a distance of 27 cm from the UV source to the cell culture dishes. Cells were irradiated with 30 mJ/cm^2^ UVR, and then collected at 4 h or 72 h after exposure. Five pairs of primary human SCC tumors with matched adjacent normal skin tissues were collected through the Molecular Pathology Shared Resource/Tissue Bank of the Herbert Irving Comprehensive Cancer Center at CUMC under IRB protocol AAAB2667. The age, gender, and race of the patients along with information on tumor stages and surgical sites of the SCC and control skin are summarized in Supplemental Table 6.

### RNA isolation and RNA-seq analysis

Total RNA was isolated from cultured keratinocytes, primary SCC tumors or adjacent normal skin tissues using the RNeasy Kit (QIAGEN, Gaithersburg, MD). All RNA samples were subsequently analyzed using an RNA 6000 nano chip (Agilent Technologies, Wilmington, DE) to confirm that the RNA integrity index was 8.0 or above. Total RNA (500 ng) from each sample was subjected to poly-A pull-down to enrich mRNAs for library preparation by using Illumina TruSeq RNA prep kit (Illumina, San Diego, CA). The resulting libraries were sequenced using Illumina HiSeq2000 at Columbia Genome Center. Sequencing reads were mapped to the human reference genome (NCBI/build37.2) using Tophat (version 2.0.4). Differentially gene expression (DGE) between irradiated and non-irradiated keratinocytes were determined using the DESeq software package^61^, with a fold change (FC) cutoff set at >2 or <0.5.

### H3K27ac ChIP-seq analysis

For ChIP-seq studies, cells were fixed with 1% (final concentration) freshly prepared formaldehyde at 37 °C for 15 min. The fixation was stopped by incubation in 125 mM (final concentration) glycine solution for 5 min at RT. Cells were washed with PBS containing proteinase inhibitor cocktail (1x final concentration), scraped and collected as cell pellets in Eppendorf tubes. Subsequent ChIP assays and sequencing were performed by Active Motif using the H3K27ac HistonePath™ Kit following standard protocols (Active Motif, Carlsbad, CA). The 75-nt sequence reads generated by Illumina sequencing were mapped to the human reference genome hg19 using the BWA algorithm with default settings. Duplicate reads were removed, and the number of aligned reads (“tags”) was adjusted to 24.2 million for each sample (by down sampling the larger data sets). These normalized tag files were used in all downstream analysis. ChIP-seq tags were extended at their 3’-ends to 200 bp. We used the model-based analysis of ChIP-seq (MACS) algorithm for peak calling to identify chromatin regions with H3K27ac tags compared to the input control^33^. Using a p-value cutoff at 1e-7, approximately 38,000 to 40,500 peaks were identified for each sample. Genes were annotated if the distance between peak-interval and gene body-interval was within 10 kb. MACS peaks (excluding promoter peaks) were used as “constituent enhancers” input into the ROSE (Rank Ordering of Super Enhancers) software to identify super enhancers (SEs). Default settings were used for the stitching (12.5 kb distance). Genes were annotated to be associated with SEs if they were within 25 kb upstream or downstream of a SE. To identify UV-induced enrichment of transcription factor (TF) motifs, we used the HOMER software for motif analysis by comparing the enhancer regions from the irradiated sample with those from the control sample.

### Whole exome-seq (WES) analysis

Genomic DNA was isolated from UV-irradiated and control samples using the Wizard Genomic DNA Purification Kit (Promega). WES was performed at the Columbia Genome Center following standard Illumina TruSeq multiplexing protocol to generate targeted number of reads with more than 85% coverage of the targeted regions by ≥15 reads and 90% covered by ≥10 reads. The resulting reads were mapped to the human reference genome hg19 using the BWA algorithm with default settings. Mapped reads were sorted and indexed using the Samtools program. Duplicate reads were marked using Picard-tools. UV-induced somatic mutations between the paired UV-4h vs. control or UV-72h vs. control were called using Samtools mpileup and bcftools with default settings. Variants with fewer than 10 reads depth were discarded from the analysis.

### Identification of UV target genes in skin carcinogenesis in the Achilles database

To identify UV target genes that are critical to skin cancer cell proliferation or survival, we queried the Achilles database with genes that were upregulated by UV. A gene was considered essential to skin cancer cell survival if their corresponding shRNAs became depleted after 40 days or 16 population doublings following shRNA infection^40^. We downloaded the raw normalized shRNA depletion score (DS) (Normalized shRNA value = log_2_ [(Raw read value for shRNA)/(Total raw read value for Replicate) × 1e6] + 1) from the Achilles database. We normalized each shRNA DS by subtracting the median DS of the negative control shRNAs, including luciferase, GFP, RFP, and LacZ in the same sample. We then performed Wilcoxon tests to compare the distribution of DS among the shRNAs targeting the same gene to the distribution of the pairwise DS of all shRNAs (the null model). If the DS of shRNAs targeting the same gene was significantly similar when compared to that of the null model (p < 0.1), we took the median DS of these shRNAs in the replicate samples as the gene-level DS for every cell line. Finally, we used the Wilcoxon test to identify genes whose DS was significantly lower in skin cancer cells than non-skin cancer cells (p < 0.05), which were considered as skin cancer-specific cancer genes. All statistical analyses were performed using the R software package.

### Immunofluorescence staining

Primary antibodies were purchased from Abcam (SLAMF7, ab202840) or One World Lab (PTGS2, TA805307_OWL; CYP24A1: 52761_OWL; GJA5: 5361_OWL). Immunofluorescence staining was performed as we previously reported^62^. Briefly, cultured cells on glass coverslips or frozen tissue sections (8 μM thickness) were fixed in 4% paraformaldehyde for 10 min or in cold acetone for 20 min. Fixed cells or tissue sections were then washed 3 times with PBS and then incubated with blocking buffer (0.1% Triton X-100 and 10% normal serum in PBS) for 1 h before being incubated with primary antibodies overnight at 4 °C in a humidified chamber. After 3 consecutive 5-min washes with PBS, cells or tissue sections were incubated with secondary antibodies for 1 h before being washed with PBS and mounted with gelvatol mounting media containing 4,6-diamidino-2-phenylindole dihydrochloride (DAPI). Images were acquired using a fluorescence confocal microscope (Zeiss, Thornwood, NY, USA).

### Statistics

Statistical analysis of each omics data set between UV-irradiated and non-irradiated keratinocytes was performed using methods included in each software package as described above. A false discovery rate <0.05 was used to control for false discoveries. The gene depletion scores between skin cancer cells and non-skin cancer cells were compared using Wilcoxon tests (R software package) and p < 0.05 was considered significant.

## Additional Information

**How to cite this article:** Shen, Y. *et al*. Epigenetic and genetic dissections of UV-induced global gene dysregulation in skin cells through multi-omics analyses. *Sci. Rep.* **7**, 42646; doi: 10.1038/srep42646 (2017).

**Publisher's note:** Springer Nature remains neutral with regard to jurisdictional claims in published maps and institutional affiliations.

## Supplementary Material

Supplemental Information
