# Supplementary material for: Epigenetic and genetic dissections of UV-induced global gene dysregulation in skin cells through multi-omics analyses
Source: Sci Rep. 2017 Feb 17;7:42646. doi: 10.1038/srep42646 (PMC5314319; doi:10.1038/srep42646)
Supplement: Supplemental Information [file srep42646-s1.pdf]

Supplementary Information

Epigenetic and genetic dissections of UV-induced global gene dysregulation in skin cells through multi-omics analyses

Yao Shen[1], Milda Stanislauskas[2], Gen Li[3], Deyou Zheng[4,5], and Liang Liu[2*]

Departments of Systems Biology[1], Dermatology[2], Biostatistics[3], Columbia University, New York, NY, USA; Departments of Genetics[4] and Neurology[5], Albert Einstein College of Medicine, New York, NY, USA.

Supplemental Table 1. Genes associated with UV-induced SNV mutations at 4h after UVR

gene	chromosome	position	locations	frequency	control	UV-4h
ZBTB2	chr6	151712026	intron	0.818181818	G	A
FAM20C	chr7	205301	intron	0.8	A	G
CDC16	chr13	115000650	intron	0.797752809	G	T
ASGR2	chr17	7004129	down1000	0.75	A	C
HPCA	chr1	33358601	intron	0.727272727	G	C
GNB5	chr15	52471606	intron	0.727272727	C	A
RBFOX1	chr16	6771873	intron	0.714285714	G	A
KCNQ2	chr20	62070160	intron	0.714285714	C	G
USP54	chr10	75289244	3utr	0.7	G	C
DENND5B	chr12	31743344	5utr	0.7	A	G
EZR-AS1	chr6	159239703	intron	0.7	G	C
EZR	chr6	159239703	intron	0.7	G	C
AK4	chr1	65614318	intron	0.689655172	A	G
GPD2	chr2	157308278	intron	0.666666667	A	G
ZNF229	chr19	44934653	codeexon	0.647058824	T	C
AGXT2L2	chr5	177659624	5utr	0.64	C	A
IPP	chr1	46191095	intron	0.636363636	G	C
GCSH	chr16	81129822	codeexon	0.636363636	G	A
IRX4	chr5	1881733	intron	0.633333333	T	C
SPATA6	chr1	48901495	intron	0.620689655	C	T
TDRD10	chr1	154474875	5utr	0.6	G	A
EFNA5	chr5	106830063	intron	0.6	C	T
AK309476	chr9	99984349	intron	0.6	A	G
CYP24A1	chr20	52782438	intron	0.583333333	T	G
AK027541	chr2	162101549	intron	0.583333333	G	A
AKNA	chr9	117107006	intron	0.583333333	A	T
SLC18A3	chr10	50818500	5utr	0.571428571	G	T
FLG	chr1	152280864	codeexon	0.571428571	A	G
FAM161A	chr2	62073918	5utr	0.571428571	C	T
RAC2	chr22	37629567	intron	0.555555556	C	T
H1F0	chr22	38201283	5utr	0.555555556	A	G
ISPD	chr7	16460893	codeexon	0.550724638	T	G
WDR5	chr9	137001593	intron	0.55	C	G
ANAPC16	chr10	73847739	intron	0.545454545	T	C
SPOCK2	chr10	73847739	intron	0.545454545	T	C
ROCK1P1	chr18	109366	3utr	0.545454545	C	A
ALLC	chr2	3751091	down1000	0.541666667	A	C
SLC24A3	chr20	19658947	intron	0.538461538	A	C
SLC24A3	chr20	19658963	intron	0.538461538	G	A

ERBB4	chr2	212389372	intron	0.538461538	C	T
MAP6D1	chr3	183535094	3utr	0.533333333	C	T
NDFIP1	chr5	141488364	5utr	0.533333333	G	C
ADAMTS2	chr5	178548136	intron	0.523809524	C	T
SMARCB1	chr22	24129129	up1000	0.52	G	T
AK056396	chr1	143153082	intron	0.5	T	A
CR936796	chr1	143153082	intron	0.5	T	A
JMJD4	chr1	227920103	codeexon	0.5	G	A
USP44	chr12	95937991	intron	0.5	C	T
SLC39A2	chr14	21469151	codeexon	0.5	T	C
VASH1	chr14	77227819	up1000	0.5	T	G
DQ588973	chr15	31089893	up1000	0.5	T	A
FAM63B	chr15	59144199	intron	0.5	A	G
LOC643542	chr18	65498815	intron	0.5	G	A
COL5A3	chr19	10120887	intron	0.5	C	G
RGPD4	chr2	108444567	intron	0.5	G	A
SYNJ2	chr6	158512154	intron	0.5	C	G
STK31	chr7	23787138	intron	0.5	G	A
DDC	chr7	50527782	intron	0.5	G	A
SEMA3C	chr7	80378319	codeexon	0.5	G	C
IFNA4	chr9	21187121	codeexon	0.5	T	A
GLRX3	chr10	131934654	5utr	0.492063492	C	G
RANBP17	chr5	170288922	5utr	0.487179487	A	G
AHNAK2	chr14	105411781	codeexon	0.473684211	G	A
INPP1	chr2	191208341	5utr	0.473684211	C	T
COL6A2	chr21	47517796	up1000	0.470588235	C	G
RNF8	chr6	37348920	intron	0.466666667	C	T
CCDC146	chr7	76924216	3utr	0.466666667	A	G
MIR548H4	chr15	69256665	intron	0.461538462	C	T
NOX5	chr15	69256665	intron	0.461538462	C	T
C1orf43	chr1	154187808	intron	0.454545455	C	T
CSPG4	chr15	75979782	codeexon	0.454545455	G	T
LRRC37A11P	chr17	37202489	intron	0.454545455	T	C
CERK	chr22	47083010	3utr	0.454545455	T	C
CERK	chr22	47083012	3utr	0.454545455	T	A
TMEM214	chr2	27259794	intron	0.454545455	G	A
ANK2	chr4	114037858	intron	0.454545455	A	G
GIGYF1	chr7	100284593	intron	0.45	C	T
DNAH11	chr7	21901566	codeexon	0.45	T	C
WAPAL	chr10	88230874	intron	0.444444444	G	A
C1orf168	chr1	57221558	intron	0.444444444	A	G
REXO1	chr19	1848628	up1000	0.444444444	A	G
CDHR3	chr7	105596532	intron	0.444444444	A	C
NUTM1	chr15	34638198	codeexon	0.4375	A	G

BTNL9	chr5	180486247	codeexon	0.432989691	G	T
IQCK	chr16	19838260	intron	0.428571429	G	A
DNAH1	chr3	52407725	intron	0.428571429	C	T
TLN2	chr15	63047778	codeexon	0.421052632	C	T
SPCS2	chr11	74660356	codeexon	0.419354839	G	A
MSS51	chr10	75186612	intron	0.416666667	G	C
DNAJC3	chr13	96343004	intron	0.416666667	G	A
NOL9	chr1	6591937	intron	0.416666667	T	C
MTOR	chr1	11190051	intron	0.411764706	A	T
EIF2S1	chr14	67849139	intron	0.411764706	A	T
OCEL1	chr19	17338099	intron	0.411764706	G	C
GPR125	chr4	22439914	codeexon	0.411764706	A	C
LOC401127	chr4	39482627	3utr	0.411764706	T	C
PRKXP1	chr15	101099378	codeexon	0.409090909	C	T
RBM20	chr10	112526712	intron	0.4	G	C
PHYH	chr10	13331412	intron	0.4	G	A
BC019904	chr11	2383401	intron	0.4	G	A
SCYL3	chr1	169862793	intron	0.4	G	A
PADI1	chr1	17557039	intron	0.4	T	C
EIF4G3	chr1	21163881	intron	0.4	C	T
HBA1	chr16	226331	up1000	0.4	G	A
CFDP1	chr16	75409474	intron	0.4	C	G
CCDC57	chr17	80108120	intron	0.4	C	T
POLR3F	chr20	18460641	intron	0.4	G	A
C20orf201	chr20	62715089	intron	0.4	G	A
OPRL1	chr20	62715089	intron	0.4	G	A
FBXW4P1	chr22	23605114	5utr	0.4	C	A
HTR3E	chr3	183819155	intron	0.4	T	C
PRRT3	chr3	9990800	codeexon	0.4	G	C
PTPRD	chr9	10060823	intron	0.4	T	C
PTPRD	chr9	10060827	intron	0.4	T	C
CXCL12	chr10	44874216	intron	0.384615385	G	A
RCBTB2	chr13	49096022	intron	0.384615385	T	C
HSP90AA1	chr14	102553255	intron	0.384615385	C	T
ZFAND6	chr15	80352332	intron	0.384615385	G	T
LOC284395	chr19	29986347	intron	0.384615385	A	C
LONRF2	chr2	100938917	5utr	0.384615385	G	C
HSPBAP1	chr3	122499441	intron	0.384615385	G	A
CAMK2D	chr4	114682714	5utr	0.384615385	G	A
PLAC9	chr10	81892696	intron	0.381818182	C	G
OR7E12P	chr11	3412900	codeexon	0.375	T	C
CYP4A22	chr1	47609407	intron	0.375	C	T
LOC100271836	chr16	21513372	intron	0.375	T	C
LOC23117	chr16	21513372	intron	0.375	T	C

TPM3P9	chr19	53945679	3utr	0.375	T	G
BTNL9	chr5	180486224	intron	0.369230769	T	G
PCSK7	chr11	117102706	intron	0.368421053	C	G
DMBT1	chr10	124349692	intron	0.363636364	A	T
LDLRAD3	chr11	35990164	intron	0.363636364	C	T
BC070093	chr11	83097971	intron	0.363636364	G	A
RARG	chr12	53615511	intron	0.363636364	T	C
AKAP13	chr15	86284342	codeexon	0.363636364	C	T
AKAP13	chr15	86284363	codeexon	0.363636364	G	A
NUDT7	chr16	77775459	intron	0.363636364	G	A
SIRPB1	chr20	1592215	codeexon	0.363636364	C	G
TPST2	chr22	26972828	intron	0.363636364	G	A
RASGRF2	chr5	80419349	intron	0.363636364	C	G
RTN4IP1	chr6	107036909	intron	0.363636364	C	T
FBXL18	chr7	5547624	intron	0.363636364	A	G
SFRP1	chr8	41152752	intron	0.363636364	T	C
CDK5RAP2	chr9	123171656	intron	0.363636364	A	G
KIAA1217	chr10	24810603	intron	0.36	C	T
AK000451	chr9	66524429	intron	0.36	C	T
AK309896	chr9	66524429	intron	0.36	C	T
GALNT18	chr11	11354346	codeexon	0.357142857	T	C
LOH12CR1	chr12	12605279	intron	0.357142857	G	T
LOH12CR1	chr12	12605305	intron	0.357142857	T	C
LOH12CR1	chr12	12605311	intron	0.357142857	A	G
KRT6A	chr12	52885190	intron	0.357142857	C	T
HERC2P2	chr15	23378091	intron	0.357142857	G	A
MYH1	chr17	10404118	intron	0.357142857	A	G
WNT9B	chr17	44929325	intron	0.357142857	C	A
QPCT	chr2	37587040	intron	0.357142857	C	A
LOC401127	chr4	39482684	3utr	0.357142857	A	C
ITGA2	chr5	52368922	intron	0.357142857	T	A
COL6A2	chr21	47517869	up1000	0.352941176	A	T
CD302	chr2	160654524	intron	0.351851852	G	T
LY75-CD302	chr2	160654524	intron	0.351851852	G	T
AK9	chr6	109909422	intron	0.35	C	T
TPM3P9	chr19	53945701	3utr	0.346153846	C	T
LINC00668	chr18	6927348	5utr	0.34375	G	A
LINC00668	chr18	6927352	5utr	0.34375	T	C
LINC00668	chr18	6927353	5utr	0.34375	G	A
PTPRD	chr9	8319806	intron	0.34375	C	T
AP4E1	chr15	51233812	intron	0.342857143	T	C
BC171896	chr1	214656740	5utr	0.342105263	A	G
SLC18A3	chr10	50818595	5utr	0.338028169	C	T
TMX2-CTNND1	chr11	57483989	intron	0.333333333	C	T

TMX2	chr11	57483989	intron	0.333333333	C	T
RARG	chr12	53615513	intron	0.333333333	C	G
ITGBL1	chr13	102305556	intron	0.333333333	A	G
HNRNPA1L2	chr13	53217408	codeexon	0.333333333	A	G
ZNF814	chr19	58385748	codeexon	0.333333333	G	A
ZNF417	chr19	58419874	3utr	0.333333333	T	C
TUBA3FP	chr22	21363240	5utr	0.333333333	C	T
SGOL1-AS1	chr3	20219885	intron	0.333333333	C	T
SGOL1	chr3	20219885	intron	0.333333333	C	T
DDX60L	chr4	169312801	intron	0.333333333	A	G
C4orf47	chr4	186357504	codeexon	0.333333333	A	G
NKAIN2	chr6	125139473	intron	0.333333333	T	G
CMAHP	chr6	25108923	codeexon	0.333333333	A	G
MDN1	chr6	90402840	codeexon	0.326086957	C	G
9-Mar	chr12	58149253	5utr	0.322580645	T	C
OR5H6	chr3	97983981	codeexon	0.318181818	G	A
LARS	chr5	145547294	intron	0.318181818	A	G
NBPF1	chr1	16913101	intron	0.315789474	C	G
FGF6	chr12	4543361	3utr	0.315789474	G	A
CDC34	chr19	531157	up1000	0.315789474	G	C
SAMD10	chr20	62609766	intron	0.314285714	C	T
ESPNP	chr1	17020048	intron	0.3125	A	G
ABHD17A	chr19	1881418	codeexon	0.3125	C	T
IGSF10	chr3	151158265	intron	0.3125	G	A
NKAIN2	chr6	125139474	intron	0.3125	G	C
COL19A1	chr6	70878198	intron	0.3125	C	T
DNAJB6	chr7	157151376	intron	0.3125	A	G
BAGE3	chr21	11022938	intron	0.307692308	C	T
TPTE	chr21	11022938	intron	0.307692308	C	T
BAGE3	chr21	11096418	intron	0.307692308	A	G
BAGE	chr21	11096418	intron	0.307692308	A	G
AX747174	chr6	39851679	3utr	0.307692308	A	G
AZGP1P1	chr7	99578381	up1000	0.307692308	T	C
SEH1L	chr18	12980604	intron	0.304347826	C	T
CLIP4	chr2	29356669	codeexon	0.304347826	A	G
FLG	chr1	152279019	codeexon	0.3	G	A
PKD1L2	chr16	81180995	5utr	0.3	T	C
DNAJC25-GNG10	chr9	114403993	intron	0.3	G	A
DNAJC25	chr9	114403993	intron	0.3	G	A
PVRL3	chr3	110793050	intron	0.297297297	G	A
FANK1	chr10	127684240	intron	0.294117647	A	G
abParts	chr16	32936350	intron	0.294117647	G	A
OR5P2	chr11	7818190	codeexon	0.291666667	C	T

ROCK1P1	chr18	109351	3utr	0.291666667	G	A
MIR1304	chr11	93466858	5utr	0.285714286	G	C
TAF1D	chr11	93466858	5utr	0.285714286	G	C
EIF2AK4	chr15	40285120	intron	0.285714286	A	G
KIAA0430	chr16	15724113	intron	0.285714286	C	T
ABCA5	chr17	67304447	codeexon	0.285714286	C	T
SPON2	chr4	1188738	intron	0.285714286	T	C
PPP1R9A	chr7	94898811	codeexon	0.285714286	G	A
AK098438	chr1	21754627	up1000	0.277777778	G	A
AK023040	chr19	23445196	intron	0.277777778	T	C
GZMK	chr5	54320700	intron	0.277777778	C	T
ANKRD12	chr18	9275542	codeexon	0.272727273	C	T
TADA1	chr1	166826773	3utr	0.269230769	C	T
FLG	chr1	152280471	codeexon	0.266666667	C	G
CHGA	chr14	93397545	intron	0.266666667	C	G
ATP2B2	chr3	10413715	codeexon	0.266666667	G	A
SPON2	chr4	1188750	intron	0.266666667	A	G
PRPH2	chr6	42672416	intron	0.266666667	A	T
SLCO2B1	chr11	74862356	5utr	0.263157895	T	C
PLEKHA5	chr12	19501256	intron	0.263157895	T	C
PCDHGA1	chr5	140710810	codeexon	0.263157895	G	A
CR936796	chr1	142688999	intron	0.261904762	T	A
SRBD1	chr2	45829286	intron	0.260869565	A	C
ESR1	chr6	152220185	intron	0.259259259	G	T
BC016143	chr1	27391101	intron	0.258064516	C	T
YAF2	chr12	42621933	intron	0.25	C	T
C3AR1	chr12	8211826	codeexon	0.25	T	C
SCNN1G	chr16	23223456	codeexon	0.25	G	T
TMPRSS9	chr19	2418031	codeexon	0.25	C	T
DPP10	chr2	116594221	intron	0.25	G	C
HGSNAT	chr8	43034584	intron	0.24	C	T
LLGL1	chr17	18144720	intron	0.238095238	C	T
SLC38A6	chr14	61482746	intron	0.235294118	G	A
SULT4A1	chr22	44230931	intron	0.235294118	T	C
YAF2	chr12	42621956	intron	0.225	G	A
GRIP1	chr12	66957328	intron	0.223684211	G	C
KIAA0922	chr4	154479430	codeexon	0.217391304	T	C
PRPH2	chr6	42672434	intron	0.217391304	C	T
TNPO3	chr7	128607384	codeexon	0.214285714	G	A
DQ583161	chr4	49563467	down1000	0.213114754	T	C
DQ590589	chr4	49563467	down1000	0.213114754	T	C
DEFB132	chr20	238540	intron	0.210526316	C	T
ADAM33	chr20	3649679	intron	0.210526316	G	T
IFT172	chr2	27680649	intron	0.210526316	A	G

IL27RA	chr19	14143434	intron	0.208333333	A	C
HBG2	chr11	5573024	intron	0.204081633	A	G
MAN1B1	chr9	139997294	intron	0.202702703	A	G
KRT3	chr12	53189446	codeexon	0.2	C	T
SNX29	chr16	12297234	intron	0.2	A	G
PALM2-AKAP2	chr9	112629858	intron	0.2	A	G
PALM2	chr9	112629858	intron	0.2	A	G
COL18A1	chr21	46930993	intron	0.192307692	C	A
SLC19A1	chr21	46930993	intron	0.192307692	C	A
TRAF3	chr14	103363623	codeexon	0.19047619	T	A
KIRREL	chr1	158020111	intron	0.1875	C	T
HS1BP3	chr2	20824596	codeexon	0.1875	C	A
DHX32	chr10	127584619	intron	0.181818182	G	A
DHX32	chr10	127584639	intron	0.181818182	G	T
NSUN6	chr10	18898986	intron	0.181818182	A	C
RASAL2	chr1	178063040	5utr	0.181818182	G	A
RASAL2-AS1	chr1	178063040	5utr	0.181818182	G	A
LOC440300	chr15	84860289	up1000	0.181818182	G	A
SF3B3	chr16	70562724	intron	0.181818182	G	C
NBAS	chr2	15701416	codeexon	0.181818182	C	T
RRBP1	chr20	17639846	codeexon	0.180722892	T	G
SCPEP1	chr17	55068388	intron	0.178571429	T	C
abParts	chr22	22730619	3utr	0.176470588	T	A
ESPNP	chr1	17023509	intron	0.175	T	C
STARD5	chr15	81605517	3utr	0.173913043	C	T
HMGCLL1	chr6	55406806	intron	0.173913043	C	T
AGAP11	chr10	88768735	codeexon	0.166666667	C	T
TMEM150B	chr19	55831576	intron	0.166666667	A	C
TMEM150B	chr19	55831577	intron	0.166666667	A	C
GALNT14	chr2	31189248	intron	0.166666667	G	T
FRG1B	chr20	29612923	intron	0.161290323	C	T
EEF2	chr19	3979340	codeexon	0.16	C	T
SLC13A1	chr7	122808598	codeexon	0.157894737	C	T
C17orf80	chr17	71232464	codeexon	0.156862745	C	G
NBPF1	chr1	16907164	intron	0.15625	C	T
GPHN	chr14	67647600	codeexon	0.153846154	C	T
NFKBID	chr19	36381388	codeexon	0.153846154	A	G
GTPBP2	chr6	43594559	intron	0.151515152	T	C
DLEC1	chr3	38101313	codeexon	0.148148148	G	T
TBX22	chrX	79286112	codeexon	0.148148148	T	C
DNM1P41	chr15	85046602	codeexon	0.147540984	C	A
RRBP1	chr20	17639850	codeexon	0.144736842	C	T
GAD2	chr10	26562588	codeexon	0.142857143	G	T
PTPRN2	chr7	157633095	intron	0.138461538	T	C

DOCK1	chr10	129160333	codeexon	0.137931034	C	G
BC080605	chr9	68415196	down1000	0.134831461	C	T
PRIM2	chr6	57254955	intron	0.134615385	G	A
CEP192	chr18	13019051	intron	0.133333333	C	A
ASPDH	chr19	51016976	intron	0.133333333	G	A
AGXT2L1	chr4	109672180	intron	0.133333333	C	T
SNAPC4	chr9	139276405	codeexon	0.133333333	G	A
RFX3	chr9	3277297	intron	0.133333333	C	A
DSPP	chr4	88537306	codeexon	0.129032258	T	C
OTOA	chr16	21747662	codeexon	0.128205128	G	A
CYP2C9	chr10	96748863	3utr	0.125	A	T
AXDND1	chr1	179418193	intron	0.12195122	T	G
NEK4	chr3	52773401	intron	0.12195122	T	C
CELA3A	chr1	22332008	codeexon	0.119047619	T	C
RTEL1	chr20	62315767	intron	0.119047619	C	T
RTEL1-TNFRSF6B	chr20	62315767	intron	0.119047619	C	T
CTNNB1	chr3	41266477	codeexon	0.117647059	C	T
AIM1L	chr1	26671625	codeexon	0.112676056	A	G
SEMA4A	chr1	156127836	intron	0.111111111	C	T
SCNN1B	chr16	23315398	intron	0.111111111	G	C
PER3	chr1	7890053	codeexon	0.109090909	G	A
MIA2	chr14	39722420	codeexon	0.108108108	G	T
MNT	chr17	2291322	codeexon	0.108108108	C	T
GINS4	chr8	41399635	3utr	0.106060606	A	G
MTMR4	chr17	56583007	intron	0.102564103	A	G

Supplemental Table 2. Genes associated with UV-induced SNV mutations at 72h after UVR

gene	chromosome	position	locations	frequency	control	UV-72h
ZNF324	chr19	58978517	5utr	0.833333333	G	A
CNFN	chr19	42893236	intron	0.642857143	T	C
JAG2	chr14	105614974	intron	0.615384615	T	C
LOC731275	chr1	243262157	intron	0.6	C	T
RHBDL3	chr17	30646333	intron	0.6	G	C
CUX1	chr7	101608918	intron	0.6	G	A
DLC1	chr8	13312149	intron	0.6	C	T
SLC35F3	chr1	234202553	intron	0.583333333	C	G
TMCC3	chr12	95034773	intron	0.571428571	T	C
DIRC3	chr2	218430506	intron	0.571428571	T	G
PFKP	chr10	3162033	intron	0.555555556	T	C
SCARB1	chr12	125325521	intron	0.555555556	C	A
ZNF365	chr10	64163201	intron	0.545454545	A	G
AK125737	chr1	17200127	intron	0.545454545	G	C
CROCC	chr1	17200127	intron	0.545454545	G	C
OBSCN	chr1	228538829	intron	0.545454545	C	T
NXN	chr17	834175	intron	0.545454545	C	G
ANK2	chr4	114037858	intron	0.545454545	A	G
DQ588973	chr15	31089893	up1000	0.538461538	T	A
PSMD1	chr2	231940362	intron	0.533333333	C	T
PRR5-ARHGAP8	chr22	45128232	codeexon	0.529411765	T	C
PRR5	chr22	45128232	codeexon	0.529411765	T	C
DL492607	chr11	113660576	3utr	0.5	A	G
SIK3	chr11	116875152	intron	0.5	A	G
DCAF6	chr1	167988529	intron	0.5	A	G
DYNLL1	chr12	120927826	intron	0.5	C	T
ITPR2	chr12	26644144	intron	0.5	T	C
JMJD4	chr1	227920103	codeexon	0.5	G	A
DBX2	chr12	45444238	intron	0.5	T	A
AK092087	chr15	35046816	up1000	0.5	T	A
GJD2	chr15	35046816	up1000	0.5	T	A
MIR3180-3	chr16	18495263	down1000	0.5	G	A
MYH1	chr17	10404118	intron	0.5	A	G
RNF112	chr17	19319374	codeexon	0.5	T	C
CAMTA1	chr1	7369470	intron	0.5	G	T
C17orf64	chr17	58503732	intron	0.5	A	C
IMPACT	chr18	22008913	intron	0.5	C	T

COL5A3	chr19	10120887	intron	0.5	C	G
DPYD-AS1	chr1	97694535	intron	0.5	C	T
DPYD	chr1	97694535	intron	0.5	C	T
CYP24A1	chr20	52789743	intron	0.5	C	G
GRIK1	chr21	31045511	intron	0.5	T	C
FBXW4P1	chr22	23605114	5utr	0.5	C	A
SERPINE2	chr2	224900339	intron	0.5	G	T
TMEM71	chr8	133769805	intron	0.5	T	G
MROH6	chr8	144654594	codeexon	0.5	G	T
ENTPD2	chr9	139944689	intron	0.5	T	C
SLCO2B1	chr11	74862356	5utr	0.473684211	T	C
TMEM247	chr2	46707886	3utr	0.473684211	G	A
SAFB2	chr19	5600370	intron	0.466666667	A	G
DDX11L9	chr15	102520232	up1000	0.461538462	C	T
EMID1	chr22	29651179	intron	0.461538462	T	C
IGSF10	chr3	151158265	intron	0.461538462	G	A
PHYH	chr10	13341900	intron	0.454545455	A	G
SYNRG	chr17	35937637	codeexon	0.454545455	T	C
DHDH	chr19	49440261	intron	0.454545455	A	G
AK127963	chr9	15069808	intron	0.454545455	A	G
OTUD6A	chrX	69283668	3utr	0.451612903	T	A
RNF8	chr6	37348920	intron	0.45	C	T
PIK3C2A	chr11	17123351	intron	0.444444444	C	T
TSPAN9	chr12	3198652	intron	0.444444444	G	A
CTH	chr1	70898646	intron	0.444444444	C	T
MGC72080	chr7	97506517	intron	0.444444444	C	T
CCDC157	chr22	30752942	5utr	0.434782609	C	A
PAG1	chr8	81905294	intron	0.434782609	G	A
TMPRSS3	chr21	43805637	codeexon	0.433333333	C	T
ELK4	chr1	205595068	intron	0.428571429	A	G
SLC45A3	chr1	205595068	intron	0.428571429	A	G
GPATCH1	chr19	33572089	5utr	0.428571429	G	C
TRAPPC12	chr2	3482796	intron	0.428571429	T	G
ITGA1	chr5	52223516	intron	0.428571429	C	A
AX746991	chr6	3287118	codeexon	0.428571429	T	C
SLC22A23	chr6	3287118	codeexon	0.428571429	T	C
MAN1B1	chr9	139994102	intron	0.423076923	G	A
CDC16	chr13	115000650	intron	0.421052632	G	T
CSPG4	chr15	75979782	codeexon	0.421052632	G	T
FRAS1	chr4	79305177	intron	0.421052632	T	G
FAM189A1	chr15	29443949	codeexon	0.416666667	A	G
LOC401127	chr4	39482627	3utr	0.416666667	T	C
INIP	chr9	115454504	intron	0.416666667	C	A
FLG	chr1	152280864	codeexon	0.4	A	G

BC043582	chr13	24593519	intron	0.4	A	G
SPATA13	chr13	24593519	intron	0.4	A	G
RCBTB2	chr13	49096022	intron	0.4	T	C
ANPEP	chr15	90342353	intron	0.4	G	A
UTS2	chr1	7949897	intron	0.4	C	T
SLC44A2	chr19	10718727	intron	0.4	C	T
ZNF101	chr19	19782507	intron	0.4	A	C
NDUFA11	chr19	5892568	3utr	0.4	G	T
GDAP1L1	chr20	42892250	intron	0.4	A	G
DPP10	chr2	116424601	intron	0.4	C	A
BID	chr22	18249905	intron	0.4	T	C
SGSM1	chr22	25320371	3utr	0.4	C	T
IQCA1	chr2	237410998	intron	0.4	T	A
FLJ16124	chr2	65790601	intron	0.4	G	A
LOC654342	chr2	91824333	intron	0.4	G	A
ANK2	chr4	114260538	intron	0.4	C	T
SLC12A7	chr5	1065683	intron	0.4	A	C
TREML4	chr6	41204094	intron	0.4	C	G
VAV2	chr9	136654553	5utr	0.4	A	C
CROCCP2	chr1	16954895	intron	0.391304348	T	C
PTPRD	chr9	8319806	intron	0.391304348	C	T
DQ572823	chr15	74365031	3utr	0.388888889	T	C
NPRL3	chr16	136888	intron	0.388888889	T	C
ABCA5	chr17	67304447	codeexon	0.388888889	C	T
NRG3	chr10	84442916	intron	0.384615385	G	A
NEGR1	chr1	72722765	intron	0.384615385	G	T
KCNQ2	chr20	62070160	intron	0.384615385	C	G
BV03S1J2.2	chr7	142495126	intron	0.384615385	G	A
TCRVB	chr7	142495126	intron	0.384615385	G	A
BV03S1J2.2	chr7	142495127	intron	0.384615385	G	T
TCRVB	chr7	142495127	intron	0.384615385	G	T
RNF123	chr3	49728818	intron	0.382352941	G	C
LLGL1	chr17	18144720	intron	0.378378378	C	T
PADI1	chr1	17557039	intron	0.375	T	C
TUBA3FP	chr22	21363240	5utr	0.375	C	T
PPP6R2	chr22	50824453	intron	0.375	T	C
RANBP17	chr5	170288922	5utr	0.375	A	G
ZNF596	chr8	182972	intron	0.371428571	T	C
BAGE3	chr21	11059877	intron	0.368421053	T	C
BAGE	chr21	11059877	intron	0.368421053	T	C
SLC25A21	chr14	37630167	intron	0.363636364	G	C
SERPINB8	chr18	61656965	down1000	0.363636364	A	C
NUDT19	chr19	33205614	down1000	0.363636364	C	T
DPP10	chr2	116594221	intron	0.363636364	G	C

RCAN2	chr6	46311223	intron	0.363636364	C	T
LOC100294362	chr17	78388947	5utr	0.357142857	G	T
AIM1	chr6	107006583	intron	0.357142857	T	A
ARID3A	chr19	968345	intron	0.352941176	C	T
ARID3A	chr19	968346	intron	0.352941176	A	G
RBKS	chr2	28006570	intron	0.352941176	C	T
BTNL9	chr5	180486247	codeexon	0.352941176	G	T
FRG1B	chr20	29612933	intron	0.351851852	G	A
ALDH3B1	chr11	67793509	codeexon	0.35	C	T
MPHOSPH9	chr12	123664438	intron	0.35	A	C
TRAP1	chr16	3724465	codeexon	0.333333333	G	C
ANKRD12	chr18	9275542	codeexon	0.333333333	C	T
HMHA1	chr19	1078994	intron	0.333333333	A	G
TMPRSS9	chr19	2418031	codeexon	0.333333333	C	T
TTC27	chr2	32865847	intron	0.333333333	C	T
CIDECP	chr3	10059717	5utr	0.333333333	G	A
FW339974	chr3	10059717	5utr	0.333333333	G	A
ATP2B2	chr3	10413715	codeexon	0.333333333	G	A
HSPBAP1	chr3	122499441	intron	0.333333333	G	A
MAP6D1	chr3	183535094	3utr	0.333333333	C	T
CMTM7	chr3	32439037	intron	0.333333333	T	C
KIAA1549	chr7	138545946	codeexon	0.333333333	C	T
NRP1	chr10	33623251	codeexon	0.322580645	G	A
FAM194B	chr13	46137837	codeexon	0.322580645	T	C
PVRL3	chr3	110793050	intron	0.322580645	G	A
PKD1L2	chr16	81180995	5utr	0.318181818	T	C
REEP1	chr2	86491245	intron	0.318181818	C	T
abParts	chr14	106452817	5utr	0.315789474	A	G
NCOA2	chr8	71036977	codeexon	0.315789474	T	C
L3MBTL3	chr6	130341389	intron	0.3125	G	A
MAGI2	chr7	77959737	intron	0.3125	A	C
COQ5	chr12	120952308	intron	0.307692308	A	C
SFSWAP	chr12	132267508	intron	0.307692308	C	G
GOLGA6L6	chr15	20740192	codeexon	0.307692308	G	A
RPTOR	chr17	78583954	intron	0.307692308	G	C
GCGR	chr17	79767602	intron	0.307692308	T	C
SH3D19	chr4	152060547	intron	0.307692308	G	A
AGGF1	chr5	76338690	intron	0.307692308	A	C
PLEKHA5	chr12	19501256	intron	0.302325581	T	C
OR7A17	chr19	14992042	codeexon	0.3	A	G
ALDH16A1	chr19	49964577	intron	0.3	G	T
BAGE3	chr21	11059845	intron	0.3	C	T
BAGE	chr21	11059845	intron	0.3	C	T
TPRXL	chr3	14106310	codeexon	0.3	T	C

TUBB2A	chr6	3157809	up1000	0.3	C	T
EPHB3	chr3	184295921	intron	0.294117647	C	T
SNX17	chr2	27598097	intron	0.291666667	T	C
KIAA1217	chr10	24783417	intron	0.285714286	C	G
TNPO3	chr7	128607384	codeexon	0.285714286	G	A
DYSF	chr2	71908167	codeexon	0.28	A	C
HPCAL1	chr2	10539355	intron	0.277777778	C	T
AGXT2L2	chr5	177659624	5utr	0.277777778	C	A
LYZL2	chr10	30915618	intron	0.275	G	A
OR7A17	chr19	14992045	codeexon	0.275	C	T
CCDC57	chr17	80109038	intron	0.272727273	C	T
FADS6	chr17	72877212	codeexon	0.266666667	C	T
SLC2A5	chr1	9107849	intron	0.266666667	G	T
MUC4	chr3	195489468	intron	0.266666667	C	G
LPAL2	chr6	160887626	5utr	0.266666667	C	A
LCN9	chr9	138556981	intron	0.266666667	C	T
IGFN1	chr1	201179068	codeexon	0.256756757	G	A
THEM4	chr1	151862656	5utr	0.25	T	C
CDC42BPB	chr14	103440282	intron	0.25	G	T
DCAF13P3	chr15	51238060	3utr	0.25	A	G
PER1	chr17	8052363	intron	0.25	T	C
ICAM4	chr19	10398859	codeexon	0.25	G	C
C2CD2	chr21	43327856	codeexon	0.25	A	G
KIAA0922	chr4	154479430	codeexon	0.25	T	C
LPAL2	chr6	160887620	5utr	0.25	C	T
GIGYF1	chr7	100284593	intron	0.25	C	T
MED14	chrX	40588602	intron	0.25	G	A
SNX29	chr16	12297234	intron	0.244444444	A	G
HBG2	chr11	5573024	intron	0.24137931	A	G
LINC00668	chr18	6927352	5utr	0.24137931	T	C
LINC00668	chr18	6927353	5utr	0.24137931	G	A
TLN2	chr15	63047778	codeexon	0.24	C	T
ALDH16A1	chr19	49964584	intron	0.238095238	A	G
C3AR1	chr12	8211826	codeexon	0.236363636	T	C
PDE6C	chr10	95388959	intron	0.235294118	T	C
DIDO1	chr20	61514496	intron	0.235294118	G	A
MCM3AP	chr21	47700939	intron	0.235294118	T	C
RTEL1	chr20	62315770	intron	0.234042553	G	A
RTEL1-TNFRSF6B	chr20	62315770	intron	0.234042553	G	A
PRPH2	chr6	42672434	intron	0.233333333	C	T
FLG	chr1	152284441	codeexon	0.227272727	G	A
CELA3A	chr1	22332008	codeexon	0.224489796	T	C
PLAC9	chr10	81892696	intron	0.222222222	C	G

LOC440300	chr15	84860289	up1000	0.222222222	G	A
OTOA	chr16	21747662	codeexon	0.21875	G	A
PLIN4	chr19	4512926	codeexon	0.21875	T	C
CARD11	chr7	2966505	intron	0.217391304	G	A
PHF2	chr9	96439004	codeexon	0.217391304	C	A
AP4E1	chr15	51233812	intron	0.216216216	T	C
LINC00668	chr18	6927348	5utr	0.214285714	G	A
BC019904	chr11	2398536	intron	0.210526316	G	C
BAGE3	chr21	11023075	intron	0.208333333	G	A
TPTE	chr21	11023075	intron	0.208333333	G	A
LOC23117	chr16	21747777	intron	0.20754717	G	A
OTOA	chr16	21747777	intron	0.20754717	G	A
CRTAC1	chr10	99664341	intron	0.2	C	T
CASP9	chr1	15850483	intron	0.2	G	A
ARHGAP5	chr14	32561340	codeexon	0.2	G	A
TCEB3B	chr18	44560300	codeexon	0.2	C	T
BRCA2	chr13	32945023	intron	0.192307692	C	A
PRIM2	chr6	57254955	intron	0.191489362	G	A
GZMK	chr5	54320700	intron	0.19047619	C	T
PRPH2	chr6	42672416	intron	0.19047619	A	T
C7orf76	chr7	96113402	intron	0.19047619	C	T
NAV1	chr1	201771084	intron	0.185185185	C	T
PLIN4	chr19	4512933	codeexon	0.181818182	C	A
SLC43A2	chr17	1486380	intron	0.173913043	T	A
PNISR	chr6	99860281	intron	0.173913043	C	A
KATNA1	chr6	149925865	codeexon	0.170212766	G	T
NBPF1	chr1	16913101	intron	0.166666667	C	G
NUDC	chr1	27272791	3utr	0.166666667	G	C
MAN1B1	chr9	139997955	5utr	0.166666667	A	G
CNTNAP2	chr7	148028756	intron	0.162790698	T	C
WRNIP1	chr6	2785341	codeexon	0.161290323	A	T
FAM166B	chr9	35562130	intron	0.161290323	G	A
CRYBA2	chr2	219855832	intron	0.16	A	T
ZNF491	chr19	11915431	5utr	0.153846154	G	A
MAN1B1	chr9	139997061	intron	0.153846154	A	G
PCDHGA10	chr5	140794674	codeexon	0.15	A	G
ABHD17A	chr19	1879995	codeexon	0.147727273	C	T
GRIP1	chr12	66957328	intron	0.146067416	G	C
TMEM54	chr1	33360395	3utr	0.142857143	G	T
MDN1	chr6	90402840	codeexon	0.142857143	C	G
KMT2C	chr7	152077092	intron	0.142857143	C	T
TNRC18	chr7	5354468	intron	0.138888889	C	T
RTF1	chr15	41772426	codeexon	0.137931034	T	C
SYNE1	chr6	152720961	intron	0.137931034	A	T

YME1L1	chr10	27408467	intron	0.135135135	T	G
RNF214	chr11	117105085	codeexon	0.133333333	G	T
GALNT10	chr5	153765954	codeexon	0.133333333	G	A
MAN1B1	chr9	139997184	intron	0.130434783	G	A
TTN	chr2	179444921	codeexon	0.129032258	C	G
MAN1B1	chr9	139998127	5utr	0.125	G	A
GBAS	chr7	56049780	intron	0.12195122	A	G
SRCAP	chr16	30723271	codeexon	0.117647059	A	G
DAPK2	chr15	64221825	intron	0.11627907	G	A
COL13A1	chr10	71637648	intron	0.111111111	C	T
LARS	chr5	145536953	intron	0.108108108	C	G
EIF1AY	chrY	22741665	intron	0.108108108	T	C
ALPPL2	chr2	233271687	intron	0.105263158	C	G
FRS2	chr12	69967861	codeexon	0.102564103	C	G
SLC35E2B	chr1	1601586	codeexon	0.1	G	T
REN	chr1	204131343	intron	0.1	A	T
ANKRD36	chr2	97779611	codeexon	0.1	A	G

Supplemental Table 3. Genes associated with common mutations between 4h and 72h

gene	chro	position	location	UV-4h frequency	UV-72h frequency	con	UV-4h/72h
CDC16	chr13	115000650	intron	0.797752809	0.421052632	G	T
KCNQ2	chr20	62070160	intron	0.714285714	0.384615385	C	G
AGXT2L2	chr5	177659624	5utr	0.64	0.277777778	C	A
FLG	chr1	152280864	codeexon	0.571428571	0.4	A	G
MAP6D1	chr3	183535094	3utr	0.533333333	0.333333333	C	T
JMJD4	chr1	227920103	codeexon	0.5	0.5	G	A
DQ588973	chr15	31089893	up1000	0.5	0.538461538	T	A
COL5A3	chr19	10120887	intron	0.5	0.5	C	G
RANBP17	chr5	170288922	5utr	0.487179487	0.375	A	G
RNF8	chr6	37348920	intron	0.466666667	0.45	C	T
CSPG4	chr15	75979782	codeexon	0.454545455	0.421052632	G	T
ANK2	chr4	114037858	intron	0.454545455	0.545454545	A	G
GIGYF1	chr7	100284593	intron	0.45	0.25	C	T
BTNL9	chr5	180486247	codeexon	0.432989691	0.352941176	G	T
TLN2	chr15	63047778	codeexon	0.421052632	0.24	C	T
LOC401127	chr4	39482627	3utr	0.411764706	0.416666667	T	C
PADI1	chr1	17557039	intron	0.4	0.375	T	C
FBXW4P1	chr22	23605114	5utr	0.4	0.5	C	A
RCBTB2	chr13	49096022	intron	0.384615385	0.4	T	C
HSPBAP1	chr3	122499441	intron	0.384615385	0.333333333	G	A
PLAC9	chr10	81892696	intron	0.381818182	0.222222222	C	G
MYH1	chr17	10404118	intron	0.357142857	0.5	A	G
LINC00668	chr18	6927348	5utr	0.34375	0.214285714	G	A
LINC00668	chr18	6927352	5utr	0.34375	0.24137931	T	C
LINC00668	chr18	6927353	5utr	0.34375	0.24137931	G	A
PTPRD	chr9	8319806	intron	0.34375	0.391304348	C	T
AP4E1	chr15	51233812	intron	0.342857143	0.216216216	T	C
TUBA3FP	chr22	21363240	5utr	0.333333333	0.375	C	T
MDN1	chr6	90402840	codeexon	0.326086957	0.142857143	C	G
NBPF1	chr1	16913101	intron	0.315789474	0.166666667	C	G
IGSF10	chr3	151158265	intron	0.3125	0.461538462	G	A
PKD1L2	chr16	81180995	5utr	0.3	0.318181818	T	C
PVRL3	chr3	110793050	intron	0.297297297	0.322580645	G	A
ABCA5	chr17	67304447	codeexon	0.285714286	0.388888889	C	T
GZMK	chr5	54320700	intron	0.277777778	0.19047619	C	T
ANKRD12	chr18	9275542	codeexon	0.272727273	0.333333333	C	T
ATP2B2	chr3	10413715	codeexon	0.266666667	0.333333333	G	A
PRPH2	chr6	42672416	intron	0.266666667	0.19047619	A	T
SLCO2B1	chr11	74862356	5utr	0.263157895	0.473684211	T	C
PLEKHA5	chr12	19501256	intron	0.263157895	0.302325581	T	C

C3AR1	chr12	8211826	codeexon	0.25	0.236363636	T	C
TMPRSS9	chr19	2418031	codeexon	0.25	0.333333333	C	T
DPP10	chr2	116594221	intron	0.25	0.363636364	G	C
LLGL1	chr17	18144720	intron	0.238095238	0.378378378	C	T
GRIP1	chr12	66957328	intron	0.223684211	0.146067416	G	C
KIAA0922	chr4	154479430	codeexon	0.217391304	0.25	T	C
PRPH2	chr6	42672434	intron	0.217391304	0.233333333	C	T
TNPO3	chr7	128607384	codeexon	0.214285714	0.285714286	G	A
HBG2	chr11	5573024	intron	0.204081633	0.24137931	A	G
SNX29	chr16	12297234	intron	0.2	0.244444444	A	G
LOC440300	chr15	84860289	up1000	0.181818182	0.222222222	G	A
PRIM2	chr6	57254955	intron	0.134615385	0.191489362	G	A
OTOA	chr16	21747662	codeexon	0.128205128	0.21875	G	A
CELA3A	chr1	22332008	codeexon	0.119047619	0.224489796	T	C

Supplemental Table 4. mRNA expression fold change (FC) of 17 histone acetyltransferases (HATs) genes and 18 histone deacetylase (HDACs) genes between UV-irradiated and control cells at 4h and 72h after UVR*

HATs	FC-4h	FC-72h		HDACs	FC-4h	FC-72h
CLOCK	0.380154	0.975132		HDAC1	1.745747	1.146615
CREBBP	0.254765	0.965611		HDAC10	1.186517	1.388792
ELP3	0.703144	0.924651		HDAC11	1.289983	1.258158
EP300	0.249285	1.253902		HDAC2	1.108064	0.82445
GTF3C4	0.351086	0.974106		HDAC3	1.346407	1.013469
HAT1	1.282991	0.64893		HDAC4	0.349709	0.930207
KAT2A	1.395039	1.105061		HDAC5	0.991799	1.225109
KAT2B	0.992872	1.190393		HDAC6	1.580243	1.144465
KAT5	0.692566	0.992226		HDAC7	0.40905	1.049525
KAT6A	0.135632	0.703423		HDAC8	1.17335	0.984343
KAT6B	0.177553	0.967992		HDAC9	0.164429	1.400943
KAT7	0.381793	0.895522		SIRT1	0.254074	0.795918
KAT8	0.825946	0.84578		SIRT2	1.677024	1.597928
NCOA1	0.369598	0.960577		SIRT3	1.141424	1.06829
NCOA2	0.163467	1.13203		SIRT4	1.089343	2.884615
NCOA3	0.258231	1.210556		SIRT5	1.194564	0.961301
TAF1	0.425664	0.993698		SIRT6	1.335281	1.235748
				SIRT7	1.569533	1.702355

*Upregulated genes are highlighted in green. Down-regulated genes are highlighted in red.

Supplemental Table 5. UV-induced SNV profiles at 4h and 72h after UVR*

4h after UVR		72h after UVR	
C_T	93	C_T	78
G_A	81	G_A	70
A_G	71	T_C	61
T_C	60	A_G	60
C_G	33	G_T	30
G_C	26	C_G	28
G_T	23	A_C	23
C_A	22	C_A	20
A_C	18	G_C	17
T_G	15	T_A	12
T_A	12	T_G	9
A_T	9	A_T	8

***Mapped sequencing reads from UVR-treated cells are normalized to sequencing reads from non-irradiated isogenic control cells to remove polymorphism.

Supplemental Table 6. SCC tumor information

Patient ID	Tissue Block Id	Patient age	Gender	Race	Pathology Class	Tumor Type	Tumor Stage	Surgical Site
**1**	16689	76	Male	White/Non-hispanic	Normal			Upper mid back
	16690	76	Male	White/Non-hispanic	Malignant, invasive	Squamous cell carcinoma	II	Upper mid back
**2**	16691	76	Male	Decline response	Normal			Oral Mucosa
	16692	76	Male	Decline response	Malignant, invasive	Squamous cell carcinoma	IVa	Oral Mucosa
**3**	17131	72	Female	White/Non-hispanic	Normal			Upper chest
	17132	72	Female	White/Non-hispanic	Malignant, invasive	Squamous cell carcinoma	II	Upper chest
**4**	17133	58	Male	White/Non-hispanic	Normal			Scalp
	17134	58	Male	White/Non-hispanic	Malignant, invasive	Squamous cell carcinoma	I	Scalp
**5**	17135	82	Male	White/Non-hispanic	Normal			Temple
	17136	82	Male	White/Non-hispanic	Malignant, invasive	Squamous cell carcinoma	II	Temple
